# Breast Milk as Route of Tick-Borne Encephalitis Virus Transmission from Mother to Infant

**DOI:** 10.3201/eid2805.212457

**Published:** 2022-05

**Authors:** Jana Kerlik, Mária Avdičová, Monika Musilová, Jana Bérešová, Roman Mezencev

**Affiliations:** Regional Public Health Authority, Banská Bystrica, Slovakia (J. Kerlik, M. Avdičová, M. Musilová, J. Bérešová);; Slovak Medical University Faculty of Medicine, Bratislava, Slovakia (M. Avdičová);; Georgia Institute of Technology, Atlanta, Georgia, USA (R. Mezencev)

**Keywords:** tick-borne encephalitis virus, TBEV, breast milk, viruses, transmission, unvaccinated mother, infant, tickborne infections, Slovakia, meningitis/encephalitis, vector-borne infections

## Abstract

Tick-borne encephalitis virus (TBEV) is transmitted mainly by tick bites, but humans can acquire infection through consuming unpasteurized milk from infected animals. Interhuman transmission of TBEV by breast milk has not been confirmed or ruled out. We report a case of probable transmission of TBEV from an unvaccinated mother to an infant through breast-feeding.

Alimentary outbreaks of tickborne encephalitis (TBE) have been caused by consuming unpasteurized milk from infected goats, sheep, and rarely also from cows (*1*). Although tick-borne encephalitis virus (TBEV) has been isolated from milk of infected animals (*2**‒**4*), interhuman transmission through breast milk has not yet been established (*5*).

At the end of May 2020, a 29-year-old woman had temporal lobe headache, neck stiffness, muscle weakness, and her temperature increased to 38.5°C. Her condition did not improve for 3–4 days, and on May 29, she was admitted to an emergency care facility. At admission, the patient reported having a transient fever 1 week before her admission that lasted several days. Her clinical evaluation led to an initial diagnosis of a neuroinfection.

Subsequently, the patient had peripheral paresis develop in the right upper limb and paresthesia in the left hand. On the second day of hospitalization, she had a generalized seizure, low peripheral O_2_ saturation of 80%, and stupor. Test results for TBEV IgM were positive for serum and cerebrospinal fluid.

Before hospitalization, the patient was breast-feeding her 8-month-old infant, including the entire period when she had clinical symptoms. On May 31, when the patient was transferred to an intensive care unit, the infant was admitted to an inpatient care unit because of fever (temperature <40°C) since the previous day. The infant did not show signs of meningeal irritation, and cerebrospinal fluid was negative for TBEV IgM. Therefore, the infant was discharged and started home care on June 4. Tests for detection of the TBEV RNA by reverse transcription PCR were not performed.

In the days after discharge, the temperature of the infant increased to 38°C. On June 11, the infant was evaluated in an emergency medical facility because of a low-grade fever and more prominent apathy. However, the infant was not admitted to an in-patient care unit and was discharged because the condition was not considered clinically serious and was thought to represent teething effects. However, on June 25, a serum specimen from the infant was collected at the office of a district pediatrician and tested for TBEV antibody. The test result was positive for TBEV IgM.

Cases of TBE in infants have been infrequently reported. However, the increasing number of cases reported more recently from several countries in Europe implies that TBE might be underreported and not exceedingly rare in infants (*6*). Some of these cases that lack a history of tick bites might have resulted from another route of transmission.

Breast-feeding is a probable route of mother-to-child transmission of TBEV because alimentary infections by unpasteurized raw milk and dairy products from infected animals have been confirmed in humans (*1**‒**4*), and mother-to-child transmission during breastfeeding has been demonstrated for Zika virus, another flavivirus that can also cross the intestinal barrier in experimental models (*7*). Depending on the animal species, TBEV is typically present in blood of infected ungulates for 1–5 days and in their milk for 2–8 days (*3*,*4*).

Conversely, this mode of transmission has not yet been conclusively demonstrated in humans. Thus far, transmission by breast milk has been suggested in a single report of serologically confirmed TBE in a mother and her breast-fed 10-day old newborn from Lithuania (*5*). We report another probable case of mother-to-infant transmission of TBEV by breast milk that is supported by clinical, epidemiologic, and serologic findings.

The incubation period for TBE is usually 7–14 days for tickborne disease but only 3–4 days for alimentary infection (*8*). These findings are consistent with the observed incubation period in the infant. The mother was hospitalized on May 29, a week after she had a transient fever. The infant had initial clinical signs of TBE on May 30.

The family lived in a disease-endemic area of ​​Banská Bystrica, Slovakia, that had the highest rate of TBE illness in this country. According to her husband, the mother consumed dairy products from an animal farm and had a tick bite 1 month before her hospitalization. He also reported lack of tick bites and denied the infant had consumed unpasteurized dairy products. The mother had not been vaccinated against TBE.

Vaccination against TBE has showed 99% efficacy (*9*) and provides short-term protection to newborns and infants through transplacental transfer of antibodies from vaccinated mothers (*10*). Because vaccination of children is recommended at 1 year of age, only nonpharmaceutical measures are available to prevent TBE in younger children.

Although alimentary transmission of TBEV from infected animals to humans by drinking raw milk has been confirmed (*1**,**4**‒**8*), mother-to-child transmission through breast milk has not (*2*). The case in this study indicates probable transmission of TBEV from an unvaccinated mother to her offspring through breast milk. This mode of transmission, if further confirmed, can have considerable implications for management of breast-feeding in unvaccinated mothers after tick bites in TBEV-endemic areas.

## References

[1] Kerlik J, Avdičová M, Štefkovičová M, Tarkovská V, Pántiková Valachová M, Molčányi T, et al. Slovakia reports highest occurrence of alimentary tick-borne encephalitis in Europe: Analysis of tick-borne encephalitis outbreaks in Slovakia during 2007-2016. Travel Med Infect Dis. 2018;26:37–42. 10.1016/j.tmaid.2018.07.001 30012472

[2] Grešíková M. Excretion of tick-borne encephalitis virus by goat’s milk [in Slovak]. Veterinársky Casopis Slovenskej Akadémie Vied. 1957;6:177–83.

[3] Grešíková M. Excretion of the tickborne encephalitis virus in the milk of subcutaneously infected cows. [in Slovak]. Acta Virol. 1958;2:188–92. 13594719

[4] Grešíková M. Recovery of the tick-borne encephalitis virus from the blood and milk of subcutaneously infected sheep. [in Slovak]. Acta Virol. 1958;2:113–9. 13545072

[5] Vaisvieliene D. TBE in Lithuania. In: Süss, J, Kahl O, editors. Proceedings of the Fourth International Potsdam Symposium on Tick-Borne Diseases; Potsdam, Germany; February 21‒22, 1997. Lengerich (Germany): Pabst Science Publishers; 1997. p. 100–13.

[6] Steffen R. Tick-borne encephalitis (TBE) in children in Europe: Epidemiology, clinical outcome and comparison of vaccination recommendations. Ticks Tick Borne Dis. 2019;10:100–10. 10.1016/j.ttbdis.2018.08.003 30241699

[7] Desgraupes S, Hubert M, Gessain A, Ceccaldi PE, Vidy A. Mother-to-child transmission of arboviruses during breastfeeding: from epidemiology to cellular mechanisms. Viruses. 2021;13:1312. 10.3390/v13071312 34372518 PMC8310101

[8] Dumpis U, Crook D, Oksi J. Tick-borne encephalitis. Clin Infect Dis. 1999;28:882–90. 10.1086/515195 10825054

[9] Heinz FX, Holzmann H, Essl A, Kundi M. Field effectiveness of vaccination against tick-borne encephalitis. Vaccine. 2007;25:7559–67. 10.1016/j.vaccine.2007.08.024 17869389

[10] Eder G, Kollaritsch H. Antigen dependent adverse reactions and seroconversion of a tick-borne encephalitis vaccine in children. Vaccine. 2003;21:3575–83. 10.1016/S0264-410X(03)00422-5 12922085

